# Molecular Identification of *Hyalomma* Ticks and Application of *Bacillus thuringiensis* Toxins as an Effective Biological Acaricide

**DOI:** 10.1155/2024/9952738

**Published:** 2024-09-11

**Authors:** Panhwer Sana Noor, Munir Ahmed, Abdul Suboor Ansari, Javaid Ali Gadahi, Shahar Bano Memon, Mansoor Tariq, Zubair Ahmed Laghari, Feroza Soomro, Bachal Bhutto, Noor-Un-Nisa Mari, Zhengli Chen

**Affiliations:** ^1^ Department of Veterinary Parasitology Sindh Agriculture University, Tandojam, Pakistan; ^2^ Laboratory of Animal Disease Model College of Veterinary Medicine Sichuan Agricultural University, Chengdu, Sichuan 611130, China; ^3^ Department of Veterinary Parasitology Shaheed Benazir Bhutto University of Veterinary and Animal Sciences, Sakrand, Pakistan; ^4^ Department of Animal Breeding and Genetics Sindh Agriculture University, Tandojam, Pakistan; ^5^ Department of Veterinary Pathology Sindh Agriculture University, Tandojam, Pakistan; ^6^ Livestock and Fisheries Department Government of Sindh, Pakistan

**Keywords:** *Bacillus thuringiensis*, bioacaricides, crystal proteins, *Hyalomma* ticks, molecular identification, Sindh, Pakistan

## Abstract

*Bacillus thuringiensis* (*B. thuringiensis*) is considered one of the most important entomopathogenic microorganisms. It produces potent toxins against insects. Therefore, the present study investigates the bioacaricidal properties of *B. thuringiensis* on the *Hyalomma* tick species. Firstly, we identify *Hyalomma* ticks based on morphological screening and molecular characterization. The cytochrome C oxidase subunit I (COX1) gene was selected for the polymerase chain reaction (PCR) analysis, which resulted in the amplification of 656 bp. The amplified products were sequenced, and the isolated (COX1) gene of ticks was submitted to the gene bank of NCBI (Accession No. OR077934.1). The nucleotide sequences were retrieved from the NCBI data bank by BLASTn analysis, which confirmed that all obtained sequences belong to genus *Hyalomma*, and multiple alignments confirmed that the sequence of *Hyalomma anatolicum* Tandojam-isolate (HA-TJ) 100% aligned with *Hyalomma analoticum* KP792577.1, *Hyalomma detritum* KP792595.1, *Hyalomma excavatum* KX911989.1, and *H. excavatum* OQ449693.1. The generated phylogenetic tree confirmed that sequences of HA-TJ COX1 clustered with a single clad of *H. analoticum*, *H. excavatum*, and *H. detritum*. The acaricidal effect of *B. thuringiensis* toxins *B. thuringiensis* spore crystal mix (*Bt*SCM) and *B. thuringiensis* crystal proteins (*Bt*cps) was evaluated against larvae and adult life stages of *Hyalomma* ticks in vitro. We applied *Bt*cps and *Bt*SCM separately with different concentrations and calculated the mortality percentage. Adult mortality was estimated at the 8th, 10th, 12th, and 15th days posttreatment and larval mortality after 24 h. During treatment of the adult life stage, at first, ticks were immersed in different concentrations of *Bt*cps and *Bt*SCM for 5 min after the treatments, and the samples were transferred to sterile containers and placed in an incubator with 80% humidity at 23°C. Furthermore, *Bt*cps produced the highest mortality on Day 15, 89 ± 1.00% at a concentration of 3000 *μ*g/mL, followed by the 12th, 10th, and 8th days produced 83 ± 1.91%, 70 ± 1.15%, and 61 ± 1.00%, respectively. *Bt*SCM produced mortality of 69 ± 1.91% on Day 15 at a concentration of 3000 *μ*g/mL, followed by the 12th, 10th, and 8th days at 57 ± 2.51%, 37 ± 1.91%, and 34 ± 2.00%. The present study revealed that *B. thuringiensis* toxins produced a significant (*p* < 0.05) increase in mortality rate in adults of *Hyalomma* ticks. Additionally, *Bt*cps and *Bt*SCM were used to treat the larval stage. The treatments were applied to calculate the mortality percentage via the Laravel packet test. At a 1500 *μ*g/mL concentration, *Bt*cps resulted in the highest mortality of 98 ± 1.15%; this was followed by 1250 *μ*g/mL, 1000 *μ*g/mL, and 750 *μ*g/mL, which produced mortalities of 76 ± 1.63%, 60 ± 1.63%, and 56 ± 1.63%, respectively. In addition, *Bt*SCM produced a mortality rate of 79 ± 2.51% at a concentration of 1500 *μ*g/mL. Furthermore, 75 ± 2.51%, 65 ± 1.91%, and 58 ± 1.15% mortality were observed at concentrations of 1250 *μ*g/mL, 1000 *μ*g/mL, and 750 *μ*g/mL, respectively. The results showed a significant (*p* < 0.05) increase in larval mortality compared to the control group. We conclude that *B. thuringiensis* toxins are applicable as a bioacaricide.

## 1. Introduction

Parasites spread globally and are considered a significant problem for livestock and other species of animals. Reportedly, 899 tick species parasitise the vertebrates including Argasidae 185 species and Ixodidae 713 species [1]. Ticks are important vectors of pathogens, mainly Crimean-Congo hemorrhagic fever (CCHF) rickettsial diseases, cowdriosis, and tick-associated dermatophilosis. These ectoparasites feed on their host's blood for varying periods, from several days to weeks. Depending on the stage, type of host, and tick species, the hard tick can suck approximately 200–600 times more blood than its body weight whereas the soft tick can consume 5–10 times [2–4].

Tick species, including *Hyalomma*, *Boophilus*, *Rhipicephalus*, and *Amblyomma*, are the primary concern for developing and developed countries due to the losses they cause. Severely infested animals reduce their body weight, become anaemic, experience severe dermatitis, weaken the hide quality by up to 20%–30%, and reduce milk production [1, 5, 6]. Ticks are also responsible for tick-bite paralysis caused by injecting a toxin by certain ticks during feeding [7, 8].

Pakistan is an agricultural country, and livestock is the backbone of its economy; recent heavy rains and migration of animals distress their health and favour the breeding of ectoparasites. The current study focused on the genus *Hyalomma*, devising more than 30 species. All these species are well adapted to hot-humid and cold weather. The present study focused on identifying ticks and applying bioacaricides from districts of Sindh, Pakistan. Previous research has looked at the incidence of tick infestation on livestock in several parts of Pakistan, and it has been recorded in a variety of hosts, including sheep, goats, and bovines; few reports mentioned that bovine tick infestation rate goes beyond 50% [9, 10].

Recent advances in molecular techniques have made it possible to identify parasites accurately. Polymerase chain reaction (PCR)–based assays have proven effective in detecting infections. Previously, identifying ticks based on their physical characteristics was challenging, especially when the specimens were damaged, engorged, or immature. Moreover, mitochondrial genomes have become important molecular markers for tick phylogeny and have significant importance in rapidly identifying tick species [11–13]. The molecular characterization of ticks is essential for taxonomic identification and phylogenetic purposes using DNA markers. These markers include nuclear (18S rRNA) and mitochondrial (12S rRNA, 16S rRNA, and COX1 genes), along with nuclear regulatory nontranslated stretches (second internal transcribed spacer [ITS2]). Additionally, 16S rRNA and C oxidase subunit I (COX1) specifically were used for tick classification at spp. level [14–18].

Parasitic resistance to multidrugs is a common problem, and various methods have been used to control the ticks; however, every control method has certain limitations. Chemicals used as acaricides against ticks have a significant effect, but it was reported that ticks have established resistance to most acaricides. This reduction in the effectiveness of a certain group of acaricides is the major contributing factor to the diversity of acaricides [19]. Multiple constraints related to ticks' control in livestock, particularly drug resistance, increase the need for new methods to control the tick population [20]. The livestock industry faces a global challenge to prevent tick infestations and tick-borne diseases in tropical and subtropical areas [21].

Therefore, researchers have directed their attention to biological control methods because of their environment-friendly characteristics. The most successful bacteria used as a bioinsecticide is *Bacillus thuringiensis*. Currently, *B. thuringiensis* is only used as a bioinsecticide in 2% of the insecticidal market. Studies have shown that it is effective against the larval stages of different insects by disrupting the tissues of their midgut and causing septicemia [22]. The insecticidal toxins produced by *B. thuringiensis* are responsible for its mode of action, although they produce additional virulence factors that contribute to insect killing [23]. During sporulation, *B. thuringiensis* has crystal inclusions containing insecticidal proteins known as Cry or Cyt toxins. A select group of toxins and proteins are specifically designed to target some insects and are known as pore-forming toxins (PFTs) produced by bacteria. These toxins are soluble in water and can change shape to insert themselves into the host membrane [22]. Moreover, *B. thuringiensis* shows potential as a biological control agent for tick control [24]. *B. thuringiensis* was responsible for killing the *Rhipicephalus microplus*–resistant strain. This approach has led to a significant reduction in the use of chemical insecticides wherever this technology has been implemented as an important alternative to tick control [25]. Therefore, the current research was conducted to evaluate the acaricidal activity of *B. thuringiensis* on *Hyalomma* ticks.

## 2. Materials and Methods

### 2.1. Ethics Statement

Animal experiments were performed following the guidelines of the Intuitional Ethics Committee, Sindh Agricultural University, Tandojam, Pakistan (Institutional Ethical Committee No. AH.06.2020, dated 5th Aug 2020).

### 2.2. Study Area

The present study is conducted in the district of Sindh, Pakistan; the livestock population of Pakistan is goats (72.2 million), cattle (44.4 million), buffalo (37.7 million), and sheep (30.1 million) [26]. Sindh province has a dry climate, with less than 200 mm yearly rainfall. The average temperature in the region ranges from 7°C to 12°C in the cold zone to above 25°C–35°C in the warm zone. During summer, ticks thrive because of the elevated humidity and temperatures. We chose random animals for the collection of ticks.

### 2.3. Sample Collection

Ticks were collected from the areas mentioned earlier. We surveyed 200 animals (ruminants) for tick collection; ticks were collected during the fall, summer, monsoon, and winter seasons in 2022. Fully engorged and partially engorged plus normal-sized ticks were sampled from the perineum, dewlap, udder, neck, ears, and skin with the help of handpicking and sterile forceps to avoid damage to mouthparts. The ticks were placed into a separate tube with soaked filter paper to supply the humidity. The collected ticks were transported to the Molecular Parasitology Laboratory, Faculty of Animal Husbandry and Veterinary Sciences, SAU, Tandojam. Upon arrival at the laboratory, ticks were washed, dried, identified, weighed, and placed in a filter paper for further procedures.

### 2.4. Conventional Identification

We followed the previously published permanent slide procedure for conventional identification. First, insert 10% KOH in a test tube, and then place the specimen. The tube was heated in a spirit lamp for 10–15 min and then transferred the specimen to a Petri dish and washed with tap water. The specimen gradually passed through 30%, 40%, 50%, 60%, 70%, 80%, and 90% alcohol for 15–20 min each. After that, it was dipped in absolute alcohol for one to several hours. Next, removed the specimen and placed it in a Petri dish containing aniline oil for dehydration. If the specimen floated, proceed with the next steps. If it did not float, it was not suitable for making a slide. Finally, placed the specimen on a slide, washed with xylol/xylene, and mounted with Canada balsam or DPX [27]. Slides were examined under 40× magnification (MRJ Microscope), and ticks were identified based on morphoanatomical characteristics [28, 29]. After that, we go for the molecular identification to confirm genus *Hyalomma*.

### 2.5. Molecular Identification

#### 2.5.1. DNA Extraction

After morphological screening, DNA was extracted with the help of a commercially available GeneJET Genomic DNA Extraction Kit (Thermo Fisher Scientific, United States) as per the manufacturer's instructions. First, the tick samples were briefly disrupted into small pieces and homogenized using the tissue homogenizer (TissueLyser LT, Qiagen, Germany). Homogenized tissue was collected in a 1.5-mL microcentrifuge tube and was resuspended in 180 *μ*L of digestion solution. After that, 20 *μ*L of proteinase K was added, and it was mixed thoroughly by vortexing to obtain a uniform suspension. The suspension was incubated at 56°C until the tissue was lysed entirely; it is very important that during the incubation, suspension was occasionally mixed by vortexing or using the shaking bath. After incubation, 20 *μ*L RNase A was added and incubated for 15 min at room temperature, and 200 *μ*L lysis buffer was added and vortexed for 10 s or until a homogenous suspension was obtained. After that, 400 *μ*L of 50% ethanol was added, mixed, and transferred to the GeneJET Genomic DNA Purification Column. The column was centrifuged at 6000 rpm for 1 min. After centrifugation, collection tubes containing filtrate were discarded, and the column was placed into a 2-mL new collection tube. Wash buffer was added to the column and centrifuged at 8000 rpm for 1 min, and the flow through was discarded. Final washing was done with Wash Buffer II and centrifuged at 8000 rpm for 3 min; the filtrate was discarded and respin the column for 1 min at 8000 rpm. Finally, 200 *μ*L of elution buffer was added to the center of the column and incubated for 2 min at room temperature. The DNA was eluted by centrifugation for 1 min at 8000 rpm. DNA was quantified with an ND-1000 spectrophotometer NanoDrop (Thermo Scientific, United States) and stored at −20°C until further use.

#### 2.5.2. PCR

The DNA was used for molecular characterization through PCR to amplify the target gene (COX1). Previously published primer of cytochrome COX1 gene (forward 5′-GGTCAACAAATCATAAAGATATTGG-3′ and reversed 5′-TAAACTTCAGGGTGACCAAAAAATCA-3) was used for the PCR to amplify 700 bp fragment of the COX1 gene [30]. PCRs were performed in a 25 *μ*L volume containing 12.5 *μ*L PCR Mix, 1 *μ*L 50 pmol of each forward and reverse primer, 1 *μ*L of genomic DNA template, and 9.5 *μ*L of deionised water. PCRs were performed in an automated thermo cycle (Applied Biosystems, United States) under the following conditions: 95°C for 5 min, 40× (95°C for 45 s, 50°C for 45 s, and 72°C for 1 min), and 72°C for 5 min. Gel electrophoresis was performed on a 1.5% agarose gel to visualize PCR products. Amplified samples were sent to Macrogen (a global digital healthcare company based on DNA sequencing) (South Korea) for sequencing.

#### 2.5.3. Sequence Analysis

The same primers used for PCR amplification were utilized for sequencing in both the forward and reverse directions for all species. Obtained sequences were confirmed by using the basic local alignment tool (https://blast.ncbi.nlm.nih.gov/Blast.cgi). Reference sequences were retrieved from the NCBI gene bank and aligned using bioinformatics tools such as Clustal X and Mega. The phylogenetic tree was constructed by using the neighbour-joining (N.J.) method.

### 2.6. Larval Hatching

Twenty-five engorged *Hyalomma* ticks were separated for egg laying. In comparison, others were used for the Adult Immersion Test (AIT). Engorged ticks were individually held in labelled glass bottles (5 × 2.5 cm) covered with muslin cloth for oviposition at 28 ± 1°C and 85 ± 5% relative humidity. After oviposition, dead females were removed to prevent fungal growth and egg contamination. The eggs were incubated at 28 ± 1°C and 85 ± 5% relative humidity for 18–25 days until they hatch into larvae. The hatched larvae were then collected for the larval packet test (LPT) [31].

### 2.7. *B. thuringiensis* Spore Crystal Mix (*Bt*SCM) and *B. thuringiensis* Crystal Protein (*Bt*cp) Isolation

The project's other half briefly cites *B. thuringiensis* culture, identification, and extraction of *Bt*SCM and *Bt*cps [32]. Pellets of *Bt*SCM and *Bt*cps were diluted in distilled water. Different dilutions of both treatments were applied to the adult and larval stages of *Hyalomma*.

### 2.8. AIT

AIT was performed on *Hyalomma* tick species according to FAO-1984 guidelines. After collection, washing, and identification, ticks were divided into six groups (A, B, C, D, E, and F), and each group comprised the same number of ticks. First, ticks were immersed in *Bt*SCM and *Bt*cp solutions diluted in distilled water for 5 min; dilution concentrations were 500, 1000, 1500, 2000, 2500, and 3000 *μ*g/mL, the control group was immersed in distilled water, and all treatments were repeated four times. After treatment, ticks were placed on Petri dishes with Whatman Filter Paper No. 1 for 24 h at room temperature; after 24 h, ticks were transferred to glass vials coated in muslin cloth, stored in desiccators at 85% relative humidity, and then incubated at 28°C in a BOD (biochemical oxygen demand) incubator. Mortality was checked twice a day for 15 days, and straightened legs of the tick were used to indicate tick paralysis. Tick paralysis was considered a lethal effect. The percentage of adult tick mortality was recorded compared to the control [31].

### 2.9. LPT

The percentage of hatched eggs was estimated visually; after 18 days of hatching, larvae were used for LPT (FAO-1984). Briefly, six different dilutions, 250, 500, 750, 1000, 1250, and 1500 *μ*g/mL of *Bt*SCM and *Bt*cps, were used to soak 3.75 cm by 8.5 cm Whatman Filter Paper No. 1 rectangles. The compound was dried in an incubator at 37°C for 30 min. Rectangles were folded in half and taped at the sides to create an open-ended package to hold tick larvae. The larvae were divided into 6 groups (A, B, C, D, E, and F), with approximately 100 larvae placed in each group, and the top of each packet was sealed with adhesive tape. There were four replicates for individual treatment. The packets were then placed in a desiccator inside a BOD incubator maintained at a temperature of 28°C and a relative humidity of 85%. After 24 h, the packets were removed, and dead larvae were counted. The control group was given distilled water [31, 33].

### 2.10. Statistical Analysis

The figures were stated as mean ± standard deviation of the mean. We compared the data variations of all the groups using one-way ANOVA. All the results in associations between groups were revealed to be diverse, and the *p* value was less than 0.05 (GraphPad Prism, United States).

## 3. Results

### 3.1. Molecular Identification of Tick Species

**Figure 1:**
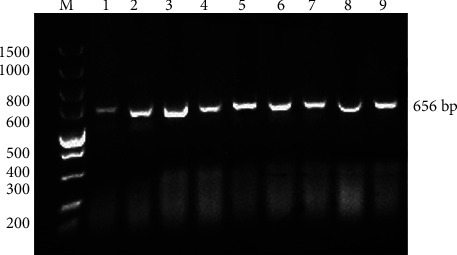
Agarose gel electrophoresis of amplified PCR product of ticks COX-1 gene showing 656 base pairs.

Before molecular identification, samples were screened morphologically to separate the unrelated group; after that, we performed molecular identification. During the present study, the COX1 gene was selected which amplified at 656 base pair regions. The PCR product from amplifying the COX1 gene was visualised using electrophoresis on an agarose gel stained with ethidium bromide (Figure 1). The results confirmed the successful gene amplification. The amplified products were sequenced for further confirmation.

#### 3.1.1. Sequence Analysis

**Figure 2:**
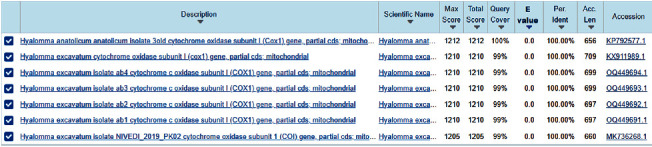
BLAST analysis for the molecular Identification of tick species.

PCR products were purified and sent to Macrogen (Korea) for sequencing, and the resulting sequence was used for sequence analysis. Obtained sequences were confirmed by using the basic local alignment tool (https://blast.ncbi.nlm.nih.gov/Blast.cgi). Results of BLAST analysis confirmed that all obtained sequences belong to *Hyalomma anatolicum*. The sequence results of the present study, denoted as *H. anatolicum* Tandojam-isolate (HA-TJ), are 100% identical to *H. anatolicum* isolate. COX1 BLAST analysis results are presented in Figure 2, and the isolated (COX1) gene of ticks was submitted to the gene bank of NCBI (Accession No. OR077934.1 HA-TJ).

**Figure 3:**
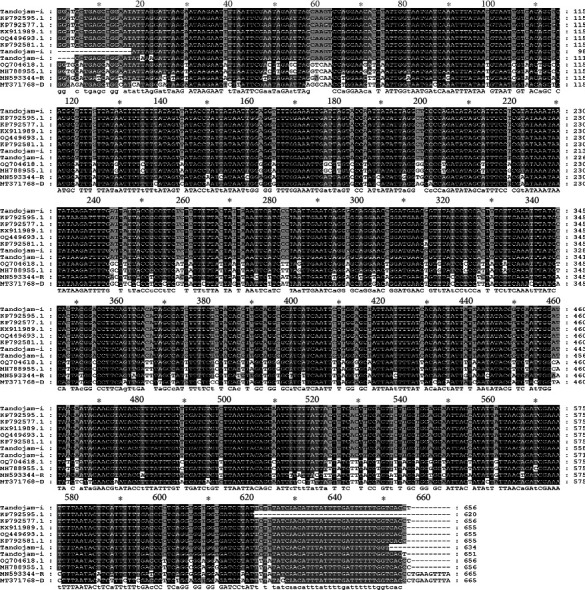
Multiple alignments of the nucleotide sequence of cytochrome C oxidase subunit I (COX1) gene of ticks. The nucleotide sequence of isolated *H. anatolicum* Tandojam-isolate (HA-TJ) aligned with the COX1 gene of other ticks retrieved from the NCBI database. *Hyalomma anatolicum* KP792577.1 (100%), *Hyalomma detritum* KP792595.1 (100%), *Hyalomma excavatum* KX911989.1 (100%), *Hyalomma excavatum* OQ449693.1 (100%), *Hyalomma anatolicum* KP792581.1 (99.85%), *Rhipicephalus microplus* OQ704618.1 (84.18%), *Rhipicephalus microplus* MH788955.1 (84.02%), *Rhipicephalus sanguineus* MN593344.1 (82.74%), and *Dermacentor auratus* MT371768.1 (81.78%).

Results of multiple alignments confirmed that the sequence of HA-TJ 100% aligned with *H. anatolicum* KP792577.1, *Hyalomma detritum* KP792595.1, *Hyalomma excavatum* KX911989.1, and *H. excavatum* OQ449693.1 (Figure 3). These findings confirmed that hard ticks collected from ruminants belong to *Hyalomma* spp.

**Figure 4:**
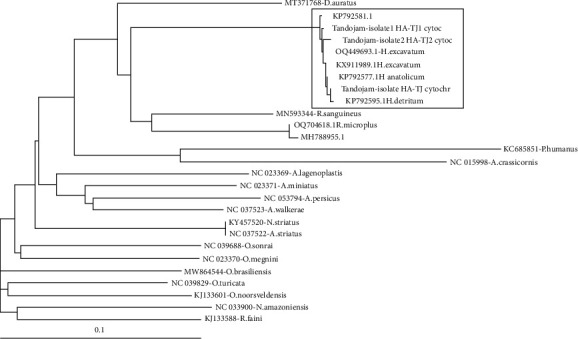
Nucleotide sequence analysis of cytochrome C oxidase subunit I (COX1) gene of ticks' species. Taxonomic relationships based on phylogenetic analysis.

Phylogenetic results indicated that all sequences of HA-TJ COX1 are closely related to *Hyalomma* species. The tree generated by phylogenetic analysis indicated that sequences of HA-TJ COX1 clustered with a single clad of *H. anatolicum*, *H. excavatum*, and *H. detritum* (Figure 4). This taxonomical relationship also confirmed that ticks prevalent in and around the localities of Sindh, Pakistan, belong to *Hyalomma* spp. (*H. anatolicum*).

### 3.2. Bioacaricidal Potential of *Bt*cps Against Adult Life Stage of *Hyalomma* Ticks

**Figure 5:**
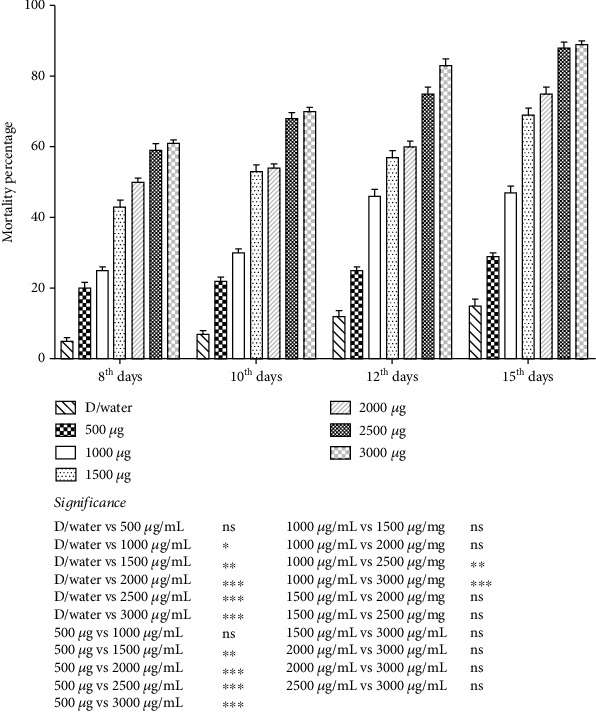
Mortality rate (percent) of adult *Hyalomma* ticks on the day at 8th, 10th, 12th, and 15th days posttreatment with varying concentrations of *Bt*cps. ^∗^*p* value 0.05. ^∗∗^*p* value 0.005. ^∗∗∗^*p* value 0.0005.

The acaricidal effect of *Bt*cps on adult *Hyalomma* ticks results in a significant (*p* < 0.05) expansion in mortality rate from the 8th to 15th days in adult *Hyalomma* ticks treated with different concentrations (Figure 5). The highest mortality was recorded on Day 15, 89 ± 1.00% at a dilution of 3000 *μ*g/mL, followed by the 12th, 10th, and 8th days at 83 ± 1.91%, 70 ± 1.15%, and 61 ± 1.00%, respectively.

### 3.3. Bioacaricidal Potential of Bacterial Solution of *Bt*SCM Against Adult *Hyalomma* Ticks

**Figure 6:**
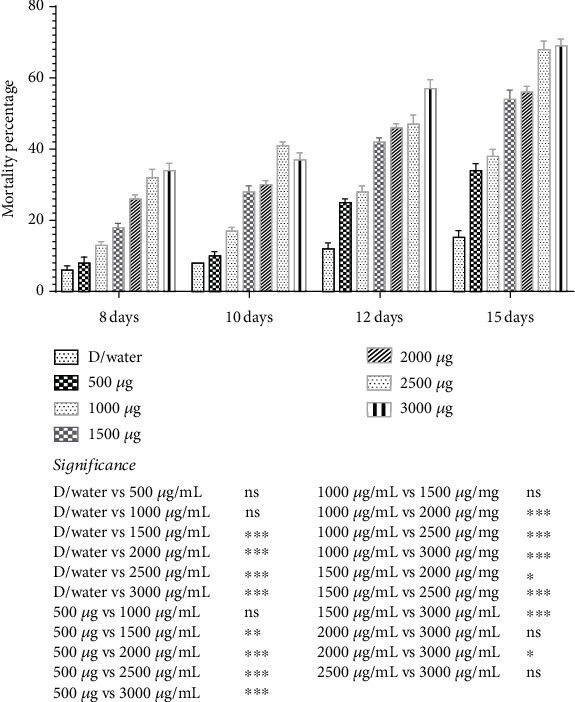
Mortality rate (percent) of adult *Hyalomma* ticks on the day at 10th, 12th, and 15th days posttreatment with varying concentrations of *Bt*SCM. ^∗^*p* value 0.05. ^∗∗^*p* value 0.005. ^∗∗∗^*p* value 0.0005.

Various concentrations of *Bt*SCM were also used against adult ticks, and we found a significant (*p* < 0.05) increase in mortality percentage from the 8th to 15th days (Figure 6). The highest mortality was recorded on Day 15, 69 ± 1.91 at a concentration of 3000 *μ*g/mL, followed by the 12th, 10th, and 8th days at 57 ± 2.51%, 37 ± 1.91%, and 34 ± 2.00%, respectively.

### 3.4. Bioacaricidal Potential of *Bt*cps Against Larval Stage of *Hyalomma* Ticks

**Figure 7:**
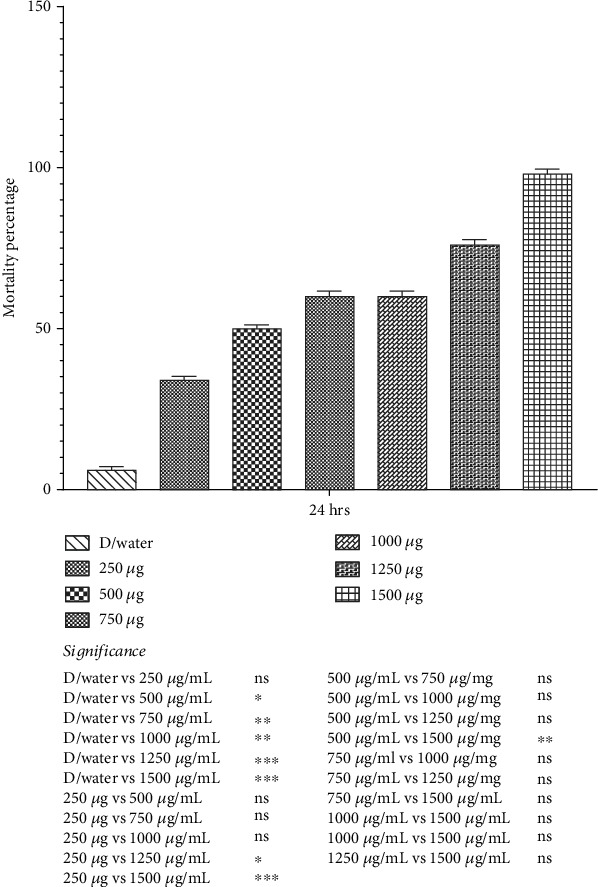
Mortality rate (percent) of larvae of *Hyalomma* ticks when treated with varying concentrations of *Bt*cps. ^∗^*p* value 0.05. ^∗∗^*p* value 0.005. ^∗∗∗^*p* value 0.0005.

*Bt*cps had dose-dependent effects on the larvae of *Hyalomma* ticks. Present results showed a significant (*p* < 0.05) increase in mortality rate within 24 h of treatment. The highest mortality recorded at the concentration of 1500 *μ*g/mL was 98 ± 1.15%, followed by 1250, 1000, and 750 *μ*g/mL at 76 ± 1.63%, 60 ± 1.63%, and 56 ± 1.63%, compared with the control group, respectively (Figure 7).

### 3.5. Bioacaricidal Potential of Bacterial Solution of *Bt*SCM Against Larval Stage of *Hyalomma* Ticks

**Figure 8:**
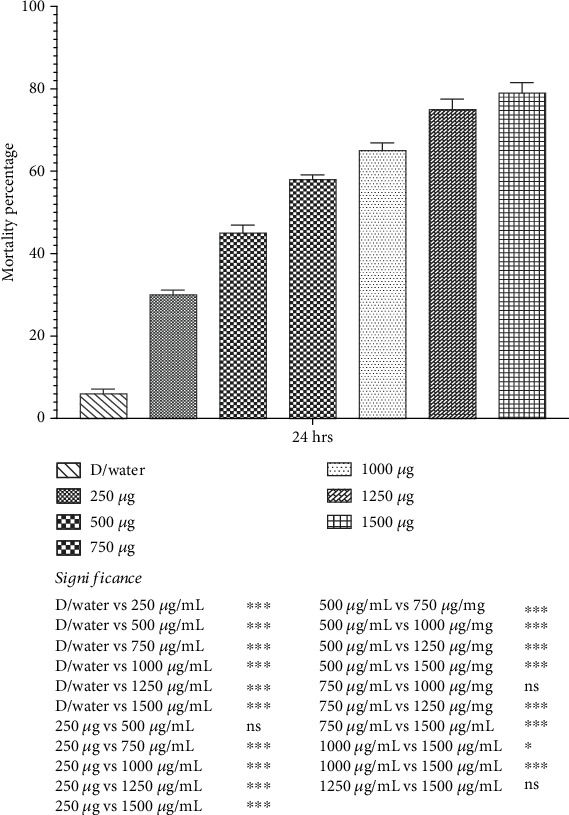
Mortality rate (percent) of larvae of *Hyalomma* ticks when treated with varying concentrations of *Bt*SCM. ^∗^*p* value 0.05. ^∗∗^*p* value 0.005. ^∗∗∗^*p* value 0.0005.

The present study's findings revealed that the *Bt*SCM produces toxicity against larvae of *Hyalomma* ticks. Results showed a significant (*p* < 0.05) increase in mortality rate when larvae of *Hyalomma* ticks were treated with different concentrations of *Bt*SCM compared to a control group (Figure 8). The highest mortality was recorded at the concentration of 1500 *μ*g/mL at 79 ± 2.51%, followed by 1250, 1000, and 750 *μ*g/mL at 75 ± 2.51%, 65 ± 1.91%, and 58 ± 1.15%.

## 4. Discussion

The major concern of the present study is the tick identification via molecular methods and the application of *B. thuringiensis* toxin as a bioacaricide. Ticks remain a significant interest globally in veterinary and medical science, as they can transmit numerous diseases and lead to infestations. The present study surveyed 200 animals (ruminants) from Sindh, Pakistan, and revealed a 38.5% tick infestation rate. Previous studies denoted that ticks are widely distributed in different ecological and geographical regions of Pakistan [9]. In addition, studies from Pakistan documented tick infestation in various hosts such as sheep, goats, and bovines [9, 10, 14, 34–37].

Previous studies mentioned 19 tick species infesting livestock in Pakistan representing three important hard ticks, *Rhipicephalus*, *Haemaphysalis*, and *Hyalomma*, and two soft ticks, *Ornithodoros* and *Argas* [9]. Present findings revealed that nucleotide sequences retrieved from the NCBI data bank by BLASTn analysis confirmed that all obtained sequences belong to genus *Hyalomma*, and multiple alignments confirmed that the sequence of HA-TJ 100% aligned with *Hyalomma analoticum* KP792577.1, *H. detritum* KP792595.1, *H. excavatum* KX911989.1, and *H. excavatum* OQ449693.1. The generated phylogenetic tree confirmed that sequences of HA-TJ COX1 clustered with a single clad of *H. analoticum*, *H. excavatum*, and *H. detritum*. Moreover, *Rhipicephalus appendiculatus* and *Amblyomma* species were also identified. Likewise, a study conducted in Faisalabad, Jhang, and Khanewal districts of Punjab, Pakistan, revealed that the prevalence of the *Hyalomma* species was significantly higher, at 61%, compared to other genera of hard ticks [38]. Moreover, another research revealed that the distribution of the *Hyalomma* genus in Iran was more than the rest of the genera, and the most frequent species belonged to this genus; the *H. anatolicum* and *H. marginatum* (the main vectors of CCHF virus) species were reported from 25 and 21 provinces, respectively [39].

Different tick infestation control measures, including acaricides, entomopathogenic fungi, essential oils, vaccines, and drugs, have benefits and drawbacks, such as causing harm to genetic material and harming the environment [40]. Therefore, scientists are more concerned about alternative ways, such as biological control of the various insects and arachnids.

Likewise, biological control with the help of the bacterium *B. thuringiensis* is an environment-friendly bacterium that is harmless to humans, animals, and plants. It can produce highly biodegradable crystal protein toxins (Cry and Cyt) during the onset of the sporulation phase [25]. *B. thuringiensis* is considered the most successful bioinsecticide, and it has gained importance globally as an alternative to chemical insecticides [41].

Previously, a study was conducted on the toxic effects of *B. thuringiensis* on mosquitoes that carry *Plasmodium* in north Burkina Faso and determined that *B. thuringiensis* toxin is a biological larvicidal against malaria mosquitoes [42].

The above study shows application of *B. thuringiensis* toxin produced a 98.5% pupa reduction in treated tubs. Furthermore, in vitro, ovicidal, and cytocidal effects of *B. thuringiensis* toxin against *Dipylidium caninum* showed that 600 *μ*g/mL was found lethal in 3 h of incubation. It provides 100% results after 8 h of treatment. Based on these findings, they proposed that *B. thuringiensis* was an accurate biological control against this zoonosis [43]. Another finding revealed that injection of *B. thuringiensis* var. *thuringiensis* H14-endotoxin in the hemocoel of hard tick (*Hyalomma dromedarii*) destroyed the granular cells and declared it abnormal [44].

Based on the evidence mentioned above regarding the acaricidal, insecticidal potential of the *B. thuringiensis* toxins, the current research was conducted to evaluate the bioacaricidal activity of *Bt*SCM and *Bt*cps on larvae and adult *Hyalomma* ticks. Our study revealed that 98% of the *Hyalomma* larvae are found dead within 24 h of treatment with *Bt*cps and 79% with *Bt*SCM at the concentration of 1500 *μ*g/mL. Moreover, during treatment of the adult life stage of *Hyalomma* ticks, at first, ticks were immersed in *Bt*cps and *Bt*SCM for 5 min, *Bt*cps produced the highest mortality on Day 15, 89 ± 1.00% at a concentration of 3000 *μ*g/mL, and *Bt*SCM produced mortality of 69 ± 1.91% on Day 15 at a concentration of 3000 *μ*g/mL. Previous studies reveal *B. thuringiensis* pathogenicity against *R. microplus*. They utilized 60 *B. thuringiensis* strains and found that four strains, GP123, GP138, GP130, and GP140, were toxic, and these strains had significant effects on ticks [25].

In another study, the acaricidal effects of *B. thuringiensis* strain GP532 on the mite *Psoroptes cuniculi* had the highest effect under experimental conditions, with an LC50 of 1.3 mg mL^−1^ and LT50 of 68 h. Furthermore, it produced histological changes in the gut of the mite [40].

Another study applied *B. thuringiensis* strains (EA3 and EA26.1) on larvae and adults of *Varroa destructor* (ectoparasites that feed on the fat body tissue of *Apis mellifera*), these toxins can control the *V. destructor* in beehives without damaging the colony, larvae, and adults of *A. mellifera* and concluded that overuse of synthetic acaricides, which may be lethal and sublethal for bees, can be reduced by using spores or toxins of *B. thuringiensis* as bio acaricides. [45]. In the current study, we used *Bt*SCM and *Bt*cps to evaluate the acaricidal effects on *Hyalomma* ticks, and our findings indicated that crystal proteins and spore crystal mix of *B. thuringiensis* had more lethal effects on tick larvae as compared to adults in a dose-dependent manner.

In another study, researchers used *B. thuringiensis* toxins as bioacaricides against various mites, such as *Acarus siro* L, *Tyrophagus putrescentiae*, *Dermatophagoides farinae* Hughes, and *Lepidoglyphus destructor*. They administered doses ranging from 0 to 100 mg g^−1^ using a feeding method. They found that the *B. thuringiensis* regimen inhibited 50% of mite growth at concentrations of 25–38 mg after 21 days [46]. These findings indicated that toxins of crystal proteins of *B. thuringiensis* are highly effective against the arachnids and support our study's results. We also applied different concentrations of (*Bt*cps) (*Bt*SCM) of *B. thuringiensis* and discovered that 1500 and 1250 *μ*g/mL were lethal to *Hyalomma* tick larvae. Similarly, 3000 and 2500 *μ*g/mL concentrations were shown to be fatal in adults.

In this study, we found that *Bt*SCM and *Bt*cps are highly effective against the larvae of *Hyalomma* ticks compared to adult ticks.

## 5. Conclusions

Our findings concluded that the *Bt*cp is highly effective against tick larvae and adult ticks. *Bt*cps are highly effective against the larvae of *Hyalomma* ticks compared to adult ticks. In the concentration of 1500 *μ*g/mL of crystal proteins, 98% of the tick larvae were found dead within 24 h of treatment with *Bt*cps and 79% in the case of *Bt*SCM. Finally, we suggested that to combat the drug resistance against various acaricidal drugs, crystal proteins of *B. thuringiensis* should be used as acaricides against multiple species of ticks.

## Data Availability

The data that support the findings of this study are available upon reasonable request.
